# Variability of soil quality indicators along with the different landscape positions of Choke Mountain agroecosystem, upper Blue Nile Basin, Ethiopia

**DOI:** 10.1016/j.heliyon.2022.e09850

**Published:** 2022-07-02

**Authors:** Demeku Mesfin, Engdawork Assefa, Belay Simane

**Affiliations:** a College of Social Science and Humanities, Wolaita Sodo University, Wolaita Sodo, Ethiopia; b College of Development Studies, Center for Environment and Development, Addis Ababa University, Addis Ababa, Ethiopia

**Keywords:** Agroecosystems, Landscape position, Soil quality indicators, Principal component analysis, Multivariate analysis of variance, Choke mountain watershed

## Abstract

Understanding the variability of soil quality indicators across topographic positions and agroecosystems (AES) is critical for improving soil fertility, productivity, and environmental sustainability. This study evaluates the variability of soil quality indicators along with the different landscape positions (upper, middle, and lower slopes) among the five AES of the Choke Mountain watershed in the upper Blue Nile Basin. A total of forty soil samples were gathered from Choke Mountain's five AES, upper, middle, and lower landscape positions. Principal component analysis (PCA) was used to determine the minimum datasets (MDS) from fourteen soil quality indicators. Using multivariate analysis of variance (MANOVA), the variability of soil quality indices among AES of the Choke, as well as variation with landscape positions, was investigated. The interaction effect of AES and topo-sequence (AES∗topo-sequence) has a substantial effect on three soil quality indicators, including soil silt content, soil pH, and available phosphorus. The highest mean value of silt content was found in the upper position of the hilly and mountainous highlands (AES 5), while the lowest was found in the lower part of the midland plain with black soil (AES 2). The highest mean pH of the soil was found in the lower part of the lowland and valley fragments (AES 1), and the lowest was in the lower position of the midland plain with brown soil (AES 3). The highest record of available P was found in AES 1's middle position and the lowest in AES 3's upper positions. AES had a significant impact on cation exchange capacity (CEC), and both AES and topography had a significant and distinct impact on organic matter (OM). Thus, the study suggests site-specific solutions to improve agricultural productivity and ease the constraints associated with each soil in each topo-sequence and AES.

## 1 Introduction

Soil is an essential part of humankind's total stock of natural resources and the foundation of food production (Buol et al., 2003). Topography, parent material, climate, biota, and time all play a role in the soil formation process (Jenny, 1945). Although soil-forming processes handle all spatial patterns, our knowledge of the determinants, particularly topographic influence, is inadequate (Amhakhian and Achimugu, 2011). Most surface processes on the planet, such as soil formation and soil development, are influenced by topography (Lawal et al., 2013). Soil parameters vary in vertical and horizontal directions due to landscape position (slope), soil-forming factors, and land use (Adegbite et al., 2019; Baskan et al., 2016). The physical and chemical qualities of soil, as well as the pattern of soil distribution throughout the landscape, are influenced by topography (Dessalegn et al., 2014; Fawole et al., 2016). The soil differs at different topographic positions because of the undulating landscape (Adegbite et al., 2019; Amhakhian and Achimugu, 2011; Atofarati et al., 2012). These affect soil-water connections, rainfall, drainage, soil erosion, textural composition, and other soil qualities, all of which have a significant impact on plant growth in a field (Atofarati et al., 2012).

In mountainous areas like Choke, soil properties vary from the summit to the toe slope along a topo-sequence. Microclimate, pedogenesis, and geologic processes all contributed to variation (Nuga et al., 2008; Ziadat et al., 2010). It has been shown that understanding the relationship between landscape and soil properties enhances the effort to improve the soil fertility, quality, and productivity. Topography-based soil quality studies are also commonly acknowledged to have a vital influence on the process that controls how soils are distributed and used in the landscape (Esu et al., 2008).

Various researchers have investigated soil-landscape position relationships elsewhere. Dessalegn et al. (2014) reported that soil organic carbon (SOC), total nitrogen (TN), available phosphorous (Ava. P) and cation exchange capacity (CEC) have increased from the upper to the lower slope positions. Miheretu and Yimer (2018) found increasing clay content, pH, organic matter, and total nitrogen, available phosphorous and percent base saturation (PBS) downslope due to soil erosion from upper slopes to the lower slopes. Bufebo et al. (2021) found that landscape position considerably affected soil quality indicators. Lower landscape positions have the highest mean value of soil organic carbon, total nitrogen, carbon to nitrogen (C/N) ratio, available P, CEC, exchangeable bases, and micronutrients. Mulugeta and Sheleme (2010) also found that soil quality indicators were altered, with varied landscape positions.

Agroecosystems (AES), a combination of agroecology, soil, terrain, and distribution of farming systems, have a significant effect on soil quality indicators (Simane et al., 2013). In a geographic location where various physiographic features like steep slopes, hilly lands, and mountainous surfaces are prevalent, topographic features, climatic elements, and vegetation cover play a significant role in influencing and characterizing soil properties (Mohammed et al., 2018). Because of high biomass production and increased soil respiration, AES accumulates high SOC stocks in cooler and moister climates, whereas warm and moist AES accumulate moderate SOC stocks (Abebe et al., 2020). According to Bewket and Stroosnijder (2003) in Ethiopia's Choke Mountain watershed and Yimer et al. (2006) Ethiopia's Bale Mountain, higher altitudes have higher carbon content than lower altitudes. Teferi et al. (2016) found higher OM content in the midland and lower OM in the upland of the Jedeb watershed. Such disparities in findings across different elevations and positions and earlier studies' limited geographical coverage showed the need for more site-specific research to accommodate the country's vast biophysical and socio-economic diversity and the Choke Mountain watershed in particular across the entire AES. This study thus will identify, select, and compare soil quality indicators of the different topo-sequences across the five AES of the Choke Mountain watershed.

The objectives of this study were to (i) identify key soil quality indicators and (ii) investigate the variability of selected soil quality indicators in different landscape positions (upper-middle-lower) across and within the five AES of Choke Mountain watersheds in the upper Blue Nile Basin, Ethiopia.

## 2 Materials and methods

### 2.1 Description of Choke Mountain watershed

**Figure 1:**
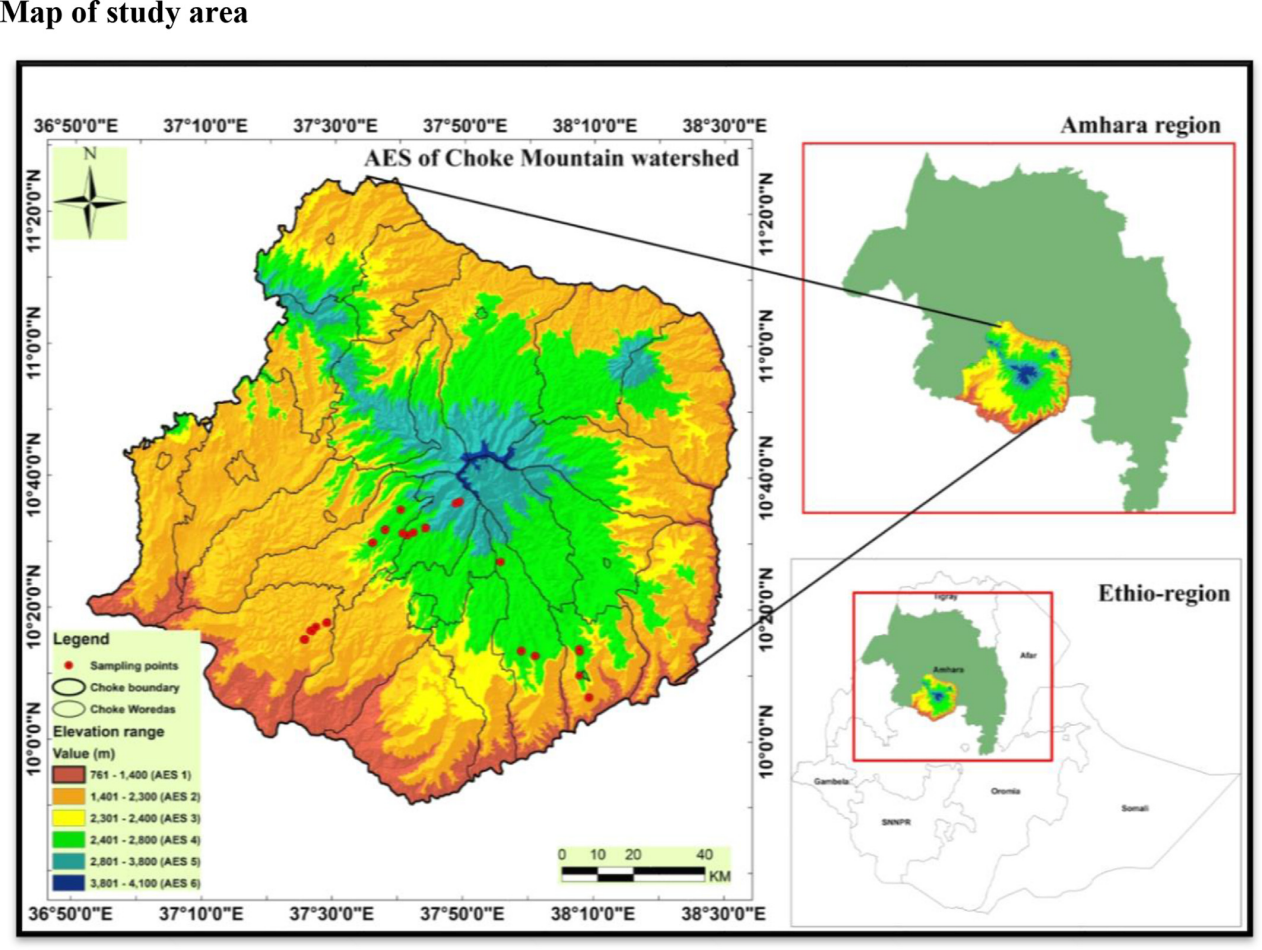
Map of Choke Mountain watershed, upper Blue Nile basin, Ethiopia.

It is a large block of highland found in central Gojjam, Amhara Regional State, Ethiopia. It is on plateaus that rise from a block of meadows and valleys and have elevations of 800–4200 m above sea level—the central peak at 100 ′ 42′ N and 370 50′ E (Figure 1). Rainfall in Choke Mountain is not evenly distributed, with average annual precipitation ranging from 600 to 2000 mm year^−1^. It exhibits local variability associated with topographic gradients. Short, occasionally violent, erosive bursts with noticeably big raindrops characterize convective precipitation episodes (Simane et al., 2013). With an average of 260–270 individuals per km^2^, the Choke Mountain AES is highly populated (Simane et al., 2013). Mixed crop-livestock agricultural systems are the primary means of livelihood, with very low inputs and outputs.

#### 2.1.1 Agroecosystems (AES) of the Choke Mountain watersheds

The Choke Mountain watershed has been divided into six AES by overlaying agroecology, soil, and farming systems (Simane et al., 2013). Since AES 6 is an Afro-Alpine on the Choke Mountain summit that ranges from 3800 to 4200, it is a protected bio-reserve and not included in the current study. The basic characteristics of the agroecosystems of Choke Mountain watershed are presented in Table 1.

**Table 1:** Characteristics of study agroecosystems of Choke Mountain watershed.

AES	Farming systems	Mean annual RF (mm)	Mean annual temp (^o^C)	Elevation Ranges (m)	Major soils	Major crops,
AES 1	Fragmented sorghum-based, extensive	<900	21–27.5	800–1400	Leptosols Cambisols	Sorghum (*Sorghum bicolor*), teff (*Eragrostis abyssinica*) Maize (*Zea mays*), haricot bean (*Phaseolus vulgaris)*
AES 2	Intensive teff-based	900–1200	11–15	1400–2300	Vertisols	Teff (*Eragrostis abyssinica*), durum wheat (*Triticum durum*), barley (*Hordeum vulgare*), chickpea (*Cicer arietinum*),
AES 3	Intensive Maize-Wheat based	900–1200	16–21	1400–2400	Nitosols Alisols	Maize (*Zea mays*), Wheat (*Triticum spp*.), Teff (*Eragrostis abyssinica*)
AES 4	Semi-intensive Wheat/barley-based	1200–1400	11–15	2400–2800	Leptosols Nitosols Alisols	Wheat *(Triticum spp.),* teff (*Eragrostis abyssinica*), barley (*Hordeum vulgare*), engido (*Avena spp.*)
AES 5	Barley/potato-based	≥1400	7.5–10	2800–3800	Leptosols Luvisols	Barley (*Hordeum vulgare*), potato (*Solamum tuberosum*), Fava bean (*Vicia fava), engido (Avena spp.)*

AES, agroecosystem; AES 1, lowland and valley fragmented; AES 2, midland plain with black soil, AES 3, midland plain with brown soil; AES 4, midland slopping lands; AES 5, hilly and mountainous highlands; RF, rainfall; temp, temperature; ^0^C, degree centigrade; mm, millimeter; m, meter.

### 2.2 Soil sample collection procedures

The writers carried out a general visual field survey, or transect walk, to view the variations across the five AES of the Choke Mountain watershed. Then, five Woredas^1^ (one from each AES) were selected randomly, and fifteen Kebeles^2^ (three from each Woreda) were selected based on their topo-unites from the Digital Elevation Model (DEM) with a 30m resolution. Three topo-unites, summit (upper part), back slope, and toe slope (lower slope) were selected from each AES.

A total of forty (5, 9, 9, 8 and 9 from AES 1, AES 2, AES 3, AES 4 and AES 5, respectively) composite soil samples were gathered using a hand auger from three slop positions in each of the five AESs of Choke watershed. The composite soil samples from five sub-samples were thoroughly mixed in a clean bucket to take 1 kg of soil from four corners and the center of a square plot of 10m by 10 m at a depth of 20 cm. This study's top 20 cm of soil depth is considered, as the depth contains the highest concentrations of many soil properties and has the most decisive response to slope difference (Miheretu and Yimer, 2018). And so is likely to be most vulnerable to soil loss (Assefa et al., 2020; Ebabu et al., 2020). Thirty-three undisturbed core soil samples were sampled from the same locations by using a sharp-edged steel cylinder (6 cm long with a 6 cm diameter) from the surface soil for BD analysis using the core method. Unwanted materials, such as dead plants, sediment deposits, furrows in the field, and organic piles, were excluded from the specific sampling sites to minimize outlying results.

### 2.3 Soil lab analysis

The soil samples were placed correctly in the plastic bags and taken to the national soil testing center for laboratory analysis. The samples were air-dried, crushed, and ground using pestle and mortar and passed through a 2 mm sieve, except for SOC and TN. A 0.5 mm sieve mesh was needed to prepare organic carbon and nitrogen (Sahlemedhin and Taye, 2000).

The sample soil was analyzed following the standard soil analytical procedures. Soil particle size distribution was determined by the Bouyoucos hydrometric method (Bouyoucos, 1962). Undisturbed soil samples were collected using a core sampler to measure bulk density (Sahlemedhin and Taye, 2000) at the soil laboratory center of Addis Ababa University, Ethiopia.

BD = oven-dry weight of soil/volume of soil

The total porosity of the soil was determined as:

*P* = (1–*BD*)/*d*) × 100

where *P* is total porosity (%), *BD* is the bulk density (g/cm^3^), and *d* is the particle density equal to 2.65 g/cm^3^ (Landon, 1991).

Soil pH was measured in a 1:2.5 soil to water suspension following the procedure outlined by Sahlemedhin and Taye (2000). The total nitrogen content was determined using the Kjeldahl method (Jackson, 1958) by oxidizing soil samples with sulfuric acid and converting the N compounds into NH4^+^ as ammonium sulfate. The Walkley and Black (1934) wet digestion method was used to determine soil carbon content, and percent SOM was calculated by multiplying percent soil organic carbon by a factor of 1.724. Phosphorus was measured according to the standard procedure of the Olsen et al. (1954) extraction method and measured by a spectrophotometer. The ammonium acetate method was used to determine CEC and exchangeable bases. An atomic absorption spectrophotometer was used to compute exchangeable Ca and Mg from the extract. A flame photometer was used to determine the exchangeable K and Na from the identical portions. Divide the sum of base cations by the cation exchange capacity multiplied by 100 to get the proportion of percent base saturation (Landon, 1991).

### 2.4 Statistical analysis

Descriptive statistics on soil quality properties were analyzed. Their normality distribution was tested with the Shapiro-Wilk test of the standardized variable to proceed with further analysis. A logarithmic transformation applied to normalize non-distributed data.

Kaiser-Meyer-Olkin (KMO) was employed to test the adequacy of sampling (Kaiser, 1974). The sample is adequate if the value of the KMO test is above the acceptable level of 0.5 (Kaiser, 1970, 1974). The strength of the association between variables is measured using Bartlett's test of sphericity. Small values of the significance level (*P* < 0.05) show that factor analysis may be helpful in data (Bartlett, 1954).

Principal component analysis (PCA) was performed on the soil quality parameters, meeting the preliminary assumption of examining the relationship among the variables by grouping them into principal components. Then the principal components (PCs) that have an eigenvalue of over one (1) are kept and selected for further analysis (Andrewsi and Carroll, 2001; Kaiser, 1960; Rezaei et al., 2006). Factor scores for each sample were computed using the regression method for analysis of variance. The correlation was conducted for factor scores to select the type of rotation. Under each PC, weighted variables (>0.7) (Teferi et al., 2016; Tesfahunegn and Gebru, 2020) were selected as soil quality indicators. Multivariate correlation coefficients were employed to check for redundancy and correlation between these highly weighted variables (Andrews et al., 2002; Vasu et al., 2016). If the variables are well-correlated *(r* > 0.60), the variables with the largest factor loading (absolute value) are preserved as an indicator (Mukherjee and Lal, 2014; Sánchez-Navarro et al., 2015; Tesfahunegn and Gebru, 2020).

Analysis of variance (ANOVA) was employed on the selected soil quality indicators to determine their significant variation in AES and topo-sequence. The significant components were then subjected to a factorial MANOVA (at *p* < 0.05). A multiple comparison test (LSD) was applied to compare the effects of the AES, topo-sequence, and their interaction on selected soil quality parameters. All the tasks were performed using the General Linear Model (GLM) procedures in SPSS (Ver.26). The magnitude of the effect of AES, topo-sequence, and their interaction was checked by looking at partial eta squared (Cohen, 1988). According to the accepted criteria of Cohen (1988), partial eta squared values ranged from 0 to 1, where 0–0.2 is a minor effect, 0.2–0.5 is medium, and over 0.5 is a large scale.

## 3 Results

### 3.1 Statistical analysis of measured soil quality indicators

The descriptive analysis revealed a numerical difference among the selected soil quality indicators in the agroecosystems of Choke Mountain watershed across the topographic positions. The analysis also showed significant statistical variation among the selected and measured soil quality parameters because of variation in AES and slop positions (Table 2).

**Table 2:** Soil quality properties (n = 40) in relation to agroecosystems and slop positions in the Choke Mountain watershed (mean ± standard error).

SQI	AES 1	AES 2	AES 3	AES 4	AES 5	Total	Upper	Middle	Lower	Total
Clay (%)	58.2 ± 2.94^a^	53.67 ± 5.29^a^	60.78 ± 2.46^ab^	53.5 ± 5.8^a^	42.67 ± 6.91^ac^	53.32 ± 2.48	42.79 ± 4.38^a^	62.62 ± 1.67^b^	55.38 ± 4.33^ab^	53.32 ± 2.48
Silt (%)	25.4 ± 2.04^a^	25 ± 2.79^a^	21 ± 1.2^ab^	26.75 ± 3.69^a^	31 ± 3.54^ac^	25.85 ± 1.36	32.57 ± 2.48^a^	21.92 ± 1.1^b^	22.54 ± 1.95^b^	25.85 ± 1.36
BD (g/cm3)	1.02 ± 0.03	1.11 ± 0.08	1.00 ± 0.02	0.98 ± 0.04	0.99 ± 0.05	1.02 ± 0.02	0.98 ± 0.04	1.08 ± 0.05	1.01 ± 0.02	1.02 ± 0.02
Porosity (%)	61.75 ± 0.95^a^	58.17 ± 2.89^a^	62 ± 0.82^b^	62.8 ± 1.62^b^	62.67 ± 1.91^b^	61.58 ± 0.82	63.08 ± 1.38^a^	59.5 ± 1.88^a^	61.82 ± 0.83^a^	61.58 ± 0.82
pH H2O	6.97 ± 0.64^a^	6.59 ± 0.18^a^	5.3 ± 0.06^b^	5.23 ± 0.13^b^	5.59 ± 0.09^b^	5.85 ± 0.14	5.56 ± 0.17^a^	5.82 ± 0.21^ab^	6.20 ± 0.31^bc^	5.85 ± 0.14
CEC (meq/100g)	41.00 ± 5.24^a^	50.75 ± 0.82^b^	27.12 ± 1.06^c^	36.27 ± 1.37^a^	39.78 ± 3.26^a^	38.85 ± 1.67	39.52 ± 2.56^b^	37.13 ± 2.87^b^	39.84 ± 3.4^b^	38.85 ± 1.67
Mg (Cmol (+)/kg)	7.84 ± 0.26^a^	13.63 ± 0.78^b^	3.04 ± 0.33^c^	2.2 ± 0.81^c^	4.67 ± 0.47^ac^	6.22 ± 0.75	5.7 ± 1.37^a^	6.15 ± 1.17^a^	6.86 ± 1.44^a^	6.22 ± 0.75
Ca (Cmol (+)/kg)	31.75 ± 10.97^a^	32.98 ± 5.12^a^	1.78 ± 0.50^b^	3.04 ± 1.38^b^	13.20 ± 2.87^c^	15.3 7 ± 2.78	1.00 ± 2.66^a^	13.45 ± 4.82^ab^	23.06 ± 6.17^bc^	15.37 ± 2.78
Ex. Na (Cmol (+)/kg)	0.07 ± 0.02^a^	0.13 ± 0.03^b^	0.03 ± 0.01^a^	0.02 ± 0.0^a^	0.05 ± 0.01^a^	0.06 ± 0.01	0.07 ± 0.02^a^	0.07 ± 0.02^a^	0.05 ± 0.01^a^	0.06 ± 0.01
Ex. K (Cmol (+)/kg)	0.61 ± 0.17^a^	0.87 ± 0.2^a^	1.06 ± 0.22^b^	0.51 ± 0.1^a^	0.42 ± 0.08^c^	0.71 ± 0.08	0.59 ± 0.12^b^	0.73 ± 0.17^b^	0.81 ± 0.14^b^	0.70 ± 0.08
PBS (%)	94.51 ± 23.29^a^	92.47 ± 9.45^a^	21.77 ± 2.60^b^	16.56 ± 6.2^b^	44.95 ± 5.37^b^	50.94 ± 6.43	37.51 ± 7.86^a^	47.12 ± 9.57^ab^	69.22 ± 14.29^bc^	50.94 ± 6.43
OM (%)	2.59 ± 0.48^a^	3.72 ± 0.88^a^	3.82 ± 0.20^a^	4.74 ± 0.90^ab^	6.92 ± 1.57^b^	4.53 ± 0.48	5.94 ± 0.88^a^	3.17 ± 0.27^ab^	4.35 ± 1.02^bc^	4.52 ± 0.48
TN (%)	0.15 ± 0.03^a^	0.22 ± 0.89^a^	0.23 ± 0.01^a^	0.28 ± 0.05^a^	0.38 ± 0.08^b^	0.26 ± 0.03	0.33 ± 0.19^a^	0.19 ± 0.02^ab^	0.25 ± 0.05^bc^	0.26 ± 0.03
Av. P (mg/kg)	11.95 ± 2.67^a^	4.8 ± 0.90^a^	3.96 ± 0.78^a^	4.69 ± 0.94^a^	5.42 ± 0.67^a^	5.62 ± 0.61	5.7 ± 0.7^ab^	4.73 ± 1.22^a^	6.43 ± 1.22^b^	5.62 ± 0.6

SQI, soil quality indicators, BD, bulk density; CEC, cation exchange capacity; Ca, calcium; Mg, magnesium; Ex. Na; exchangeable sodium; Ex. K, exchangeable potassium; PBS, percent base saturation; OM, organic matter; TN, total nitrogen; Ava. P, available phosphorus; values followed by the same letter along each row are not significantly different at *p* < 0.05 among agroecosystems and slop positions.

### 3.2 Grouping and selecting of minimum data set

The Kaiser-Meyer-Olkin (KMO) measure tested the sampling adequacy for factor analysis, KMO = 0.62, showing that it is above the acceptable level of 0.5. Bartlett's test of sphericity had an associated *P* value of less than 0.001, which was highly significant (x^2^ (91) = 655.91, *P* = 0.000), which justified continuing and performing accurate factor analysis. Four components with eigenvalues above the Kaiser's criterion of 1 were kept.

In combination, principal components (PCs) explained 84.35% of the variability in the measured soil properties (Table 3). The first principal component (PC 1) accounts for nearly 39% of total variance with high loadings (>0.7). Component-1 was correlated with variables, including clay and silt content, BD, porosity, OM, and TN.

**Table 3:** The factor loadings after Varimax rotation and communality estimates (factor loadings >0.7 appear bold, and the underlined variables were kept as MDS) (n = 40).

Soil Quality Indicators	PC 1	PC 2	PC PC 3	PC 4	Communality Estimates
Clay (%)	**-0.829**	0.006	-0.347	0.258	0.875
Silt (%)	**0.773**	-0.016	0.365	-0.344	0.849
BD (g/cm^3^)	**-0.766**	0.136	0.074	-0.541	0.904
Porosity (%)	**0.768**	-0.133	-0.057	0.547	0.91
pH H_2_O	-0.294	**0.786**	0.467	0.092	0.931
CEC (meq/100g)	0.348	**0.822**	-0.041	-0.246	0.859
Ca (Cmol (+)/kg)	-0.107	**0.937**	0.247	-0.024	0.951
Mg (Cmol (+)/kg)	-0.297	**0.899**	-0.079	-0.136	0.922
Ex. Na (Cmol (+)/kg)	0.086	0.637	-0.446	-0.11	0.625
Ex. K (Cmol (+)/kg)	-0.08	-0.093	0.112	**0.708**	0.529
PBS (%)	-0.242	**0.871**	0.358	0.033	0.946
OM (%)	**0.951**	-0.122	-0.106	-0.024	0.931
TN (%)	**0.937**	-0.102	-0.164	0.02	0.916
Ava. P (mg/kg)	0.097	0.222	**0.773**	0.085	0.663
Initial eigenvalues	5.458	3.712	1.411	1.228	
Variance (%)	38.988	26.518	10.076	8.772	
Cumulative (%)	38.988	65.506	75.582	84.354	

PC, principal components; BD, bulk density; CEC, cation exchange capacity; Ca, calcium; Mg, magnesium; Ex. Na; exchangeable sodium; Ex. K, exchangeable potassium; PBS, percent base saturation; OM, organic matter; TN, total nitrogen; Ava. P, available phosphorus.

Note: The effects of AES, topo-sequence, and interaction of the two (AES x topo-sequence) on PC 4 are not significant (*P* < 0.05). Therefore, PC 4 (exchangeable K) was not analyzed in MANOVA and discussion part.

The second PC explained 26.5% of the variance with high loadings and positively correlated pH, CEC, Ca, Mg, and PBS variables. Component-3 accounted for 10% of the total variance with high loadings from available P with a positive correlation. Component-4 explained 8.77% of the total variance with high loading from exchangeable K (Table 3).

The proportional importance of the soil quality characteristic is determined by the indicator's communality score. Communalities calculated the proportion of each soil indicator's variance that was explained by the four components. The four components accounted for over 93% of the variation in soil pH, Ca, PBS, and OM.; >90% in BD, porosity, Mg, and total nitrogen; > 84% in CEC, silt, and clay content; > 62% in exchangeable Na and available P, and the last and most negligible contribution was explained by > 52% in exchangeable K (Table 3).

Correlation coefficients show that there were variables in PC 1 that were well-correlated (r > 0.60) (Table 4). Thus, variables with the highest absolute value factor loading (silt and OM) were kept as the minimum dataset from PC 1, naming PC 1 as "root penetration and water retention."

**Table 4:** Pearson correlation coefficients of soil quality indicators (n = 40).

	SQIs	BD	Porosity	Silt	Clay	pH H2O	CEC	Ca	Mg	Ex. Na	Ex. K	PBS	OM	TN
	BD (g cm^−3^)	1												
	Porosity (%)	-.998∗∗	1											
	Silt (%)	-.381∗	.386∗	1										
	Clay (%)	.416∗	-.422∗	-.902∗∗	1									
	pH H2O	0.295	-0.284	-0.06	0.056	1								
	CEC (meq/100g)	0.02	-0.017	0.294	-.333∗	.532∗∗	1							
	Ca (cmol kg^−1)^	0.262	-0.257	-0.011	-0.001	.897∗∗	.760∗∗	1						
	Mg (cmol kg^−1^)	.423∗	-.424∗	-0.105	0.078	.740∗∗	.728∗∗	.815∗∗	1					
	Ex. Na (cmol kg^−1^)	0.03	-0.034	0.078	-0.02	0.303	.519∗∗	.400∗	.604∗∗	1				
	Ex. K (cmol kg^−1^)	-0.138	0.148	-0.125	0.029	0.202	0.035	0.219	0.171	0.141	1			
	PBS (%)	0.287	-0.281	-0.041	0.049	.948∗∗	.615∗∗	.953∗∗	.825∗∗	.377∗	0.223	1		
	OM (%)	-.688∗∗	.690∗∗	.627∗∗	-.715∗∗	-.340∗	0.282	-0.136	-0.241	0.177	0.074	-0.252	1	
	TN (%)	-.709∗∗	.709∗∗	.568∗∗	-.667∗∗	-.348∗	0.266	-0.143	-0.247	0.177	0.053	-0.266	.977∗∗	1
	Ava. P (mg/kg)	-0.073	0.091	0.208	-0.164	.537∗∗	0.263	.439∗∗	0.151	0.214	0.123	.440∗∗	0.022	0.009

SQIs, soil quality indicators; BD, bulk density; CEC, cation exchange capacity; Ca, calcium; Mg, magnesium; Ex. Na; exchangeable sodium; Ex. K, exchangeable potassium; PBS, percent base saturation; OM, organic matter; TN, total nitrogen; Ava. P, available phosphorus; ∗∗ Significant at the 0.01 level (2-tailed). ∗ Significant at the 0.05 level (2-tailed).

The correlation coefficients of variables included under the principal component-2 revealed that percent base saturation (PBS) was well-correlated with pH, Ca, and Mg, and Mg was well-correlated with pH, CEC, and Ca (Table 4). Hence, only pH and CEC were kept as a minimum dataset from PC 2 and named ''nutrient retention and availability". Available P was the only high loading variable in principal component-3 (Table 4) and was kept as MDS of soil quality indicators from PC 3 and named as "Macro-nutrient". Thus, five (silt content, PH, CEC, OM and available P) of the total soil quality indicators were selected as a minimum dataset.

### 3.3 MANOVA assumption test

Multivariate normality of dependent variables was satisfied, which was checked by a statistically non-significant *(P* > 0.05) result of the Shapiro-Wilk test. Linearity and multicollinearity assumptions were also met. Homogeneity of variance was examined using independent Levenes tests for each dependent variable, and all dependent variables were found to have a statistically non-significant (*P* > 0.05) influence. The non-significant result shows that each dependent variable has the same variance across treatment levels (AES and topo-sequence). Box's test (Box's M = 42.069, *P* = 0.019) of statistically non-significant *(P* > 0.001) (Malo, 2016) results satisfied the assumption of homogeneity of variance-covariance matrices. Meaning the dependent variables' covariance matrices were equal across the levels of the treatments. Bartlett's test of sphericity was statistically significant (χ2 = 69.566, *P* = 0.000), satisfying the strength of the relationship among dependent variables.

### 3.4 Multivariate interaction and main effects on indicator variability

The non-significant Box's M test suggests the most robust and commonly used statistics, Wilks' lambda, to interpret multivariate tests. Wilks' lambda criteria show that the multivariate main effects of AES were statistically significant (Λ = 0.007, F (24, 50.05) = 6.415, *P* = 0.000, η 2 = 0.706) (Table 5). The result shows that AES significantly affects the combined dependent variables. Multivariate main effects of topo-sequence were also significant (Λ = 0.051, F (12, 28) = 8.038, *P* = 0.000, η 2 = 0.775). MANOVA results also show the statistically significant interaction effects of AES and topo-sequence on combined dependent variables (Λ = 0.004, F (42, 69.118) = 3.72, *P* = 0.000, η 2 = 0.603) (Table 5).

**Table 5:** Result of MANOVA test for the top (0–20cm) soil quality indicators: the data from five AES of the Choke watershed along the three topo-sequences was pooled together for this analysis (n = 40).

Effect		Value	F	df	Error df	Sig.	Eta Squared *(η*^*2*^*)*
AES	Pillai's Trace	2.487	4.656	24	68	.000	0.622
	Wilks' Lambda	0.007	6.415	24	50.05	.000	0.706
	Hotelling's Trace	13.768	7.171	24	50	.000	0.775
	Roy's Largest Root	8.834	25.029	6	17	.000	0.898
Topo-sequence	Pillai's Trace	1.526	8.049	12	30	.000	0.763
	Wilks' Lambda	0.051	8.038	12	28	.000	0.775
	Hotelling's Trace	7.364	7.977	12	26	.000	0.786
	Roy's Largest Root	5.153	12.881	6	15	.000	0.837
AES ∗ Topo-sequence	Pillai's Trace	2.674	2.182	42	114	.001	0.446
	Wilks' Lambda	0.004	3.72	42	69.118	.000	0.603
	Hotelling's Trace	20.741	6.091	42	74	.000	0.776
	Roy's Largest Root	15.445	41.921	7	19	.000	0.939

AES, agroecosystem; *η*^*2*^*,* partial eta squared: The effect of topo-sequence, agroecosystem, and interaction effect was significant and large when the value of *P* < 0.05 and Eta Squared (η^2^) > 0.5.

A separate univariate analysis of variance with the selected soil quality indicators (dependent variables) was conducted as a follow-up test for MANOVA. It was conducted to find a significant group difference for each dependent variable (Table 6).

**Table 6:** Univariate test results (n = 40).

Source	Dependent Variable	*F*	Sig.	Partial Eta Squared
AES	pH H_2_O	31.259	.000	0.868
	CEC	9.286	.000	0.662
	Silt	6.775	.001	0.588
	OM	4.687	.008	0.497
	Ava. P	6.153	.002	0.564
Topo-sequence	pH H_2_O	16.067	.000	0.628
	CEC	0.977	.395	0.093
	Silt	22.979	.000	0.708
	OM	3.442	.048	0.178
	Ava. P	7.961	.003	0.456
AES ∗ Topo-sequence	pH H_2_O	10.745	.000	0.798
	CEC	1.439	.248	0.346
	Silt	8.509	.000	0.758
	OM	1.869	.132	0.408
	Ava. P.	5.544	.001	0.671

AES, agroecosystem; CEC, cation exchange capacity; OM, organic matter; Ava. P, available phosphorus.

#### 3.4.1 AES and topo-sequence interaction effect

As depicted in Table 5, the interaction effect of AES and topo-sequence (AES∗topo-sequence) was statistically significant and the effects of the magnitude was high on silt content (*F* = 8.509, *P* = 0.000, *η*^*2*^ = 0.758), pH (*F* = 10.745, *P* = 0.000, *η* ^*2*^ = 0.798) and available P (*F* = 5.544, *P* = 0.001, *η* ^*2*^ = 0.671).

The highest mean value of silt content was found in the top portion of AES 5, while the lowest silt content was found in the lower part of AES 2. The lowest mean pH of the soil (mean = 4.84) was recorded in the upper portion of AES 4, and the highest mean pH of the soil (mean = 8.05) was recorded in the lower part of AES 1. There was also an interaction effect of topo-sequence and AES on available P, with the highest record in AES 1's middle position and the lowest in AES 3's top slope (Table 7).

**Table 7:** Interaction effects of topo-sequence and AES on the three soil quality indicators (mean ± Standard error) (n = 40).

		Agroecosystems of Choke watershed				
Soil property	Topo-sequence	AES 1	AES 2	AES 3	AES 4	AES 5
Silt (%)	Upper slope	21 ± 2.94	33.66 ± 2.40	22.33 ± 2.40	39 ± 2.40	43 ± 2.40
	Middle slope	25 ± 4.16	24.33 ± 2.40	21 ± 2.40	20.33 ± 2.40	21 ± 2.40
	Lower slope	30 ± 2.94	17 ± 2.40	19.66 ± 2.40	18 ± 2.94	29 ± 2.40
pH H2O	Upper slope	5.41 ± 0.2	6.6 ± 0.16	5.36 ± 0.16	4.84 ± 0.16	5.51 ± 0.16
	Middle slope	7.95 ± 0.28	6.09 ± 0.16	5.32 ± 0.16	5.51 ± 0.16	5.66 ± 0.16
	Lower slope	8.05 ± 0.2	7.09 ± 0.16	5.21 ± 0.16	5.39 ± 0.1	5.60 ± 0.16
Ava.P. (mg/kg)	Upper slope	6.00 ± 1.62	7.19 ± 1.32	3.00 ± 1.32	4.86 ± 1.32	7.50 ± 1.32
	Middle slope	18.69 ± 2.29	3.85 ± 1.32	3.70 ± 1.32	3.185 ± 1.32	3.52 ± 1.32
	Lower slope	14.52 ± 1.62	3.34 ± 1.32	5.15 ± 1.32	6.684 ± 1.62	5.24 ± 1.32

AES 1, lowland and valley fragmented; AES 2, midland plain with black soil, AES 3, midland plain with brown soil; AES 4, midland slopping lands; AES 5, hilly and mountainous highlands; Ava. P, available phosphorus.

#### 3.4.2 Main effects of AES and topo-sequence on indicators' variability

The main effect of AES was statistically significant for CEC (*F* = 9.286, *P* = 0.000, *η* ^*2*^ = 0.868) and OM (*F* = 4.687, *P* = 0.008, *η* ^*2*^ = 0.497). OM was also significantly affected by the main effects of landscape position (*F* = 3.442, *P* = 0.048, *η* ^*2*^ = 0.17) (Table 6).

## 4 Discussions

This study assessed the variability of soil quality indicators along the various topo-sequences at AES of the Choke Mountain watershed by using factorial MANOVA. Silt content, pH, available P, OM, and CEC, are the soil quality indicators related to AES and the landscape position of the watershed. Because the last component had no significant effects, the change in soil attribute of the corresponding component could not be explained by either AES or topo-sequence. A critical review by Bünemann et al. (2018) also recommended that, with the PCA technique, the number of indicators selected typically ranges between 6 and 8. This study's findings are like those of (Teferi et al., 2016). They investigated silt content, bulk density, soil pH, and organic matter as important soil quality indicators for evaluating agroecology or land use.

### 4.1 The soil silt content

The result shows that the silt content of the soil increases with the increasing slope of the watersheds. According to this, a study done in Thailand's Wat Chan watershed found a positive, significant connection between topsoil silt and SOM as slope angle increased (Sangchyoswat and Yost, 2002). These links could be because of the structural aggregation of silt and the accumulation of SOM in the Choke Mountain watershed's wet and high-altitude zones.

The silt content of the soil fraction also showed significant variation among the five agroecosystems (AES) and landscape positions (*P* < 0.05). The multivariate effect size statistics revealed the main effects of AES (*η*^*2*^ = 0.588) and landscape position (*η*^*2*^ = 0.708) were significant (Table 6). The AES of Choke significantly affects the silt content of the soil, and the hilly and mountainous highland (AES 5) accounts for the highest value (mean = 31%) of silt (Table 2). It could be because of the highest value of the OM in AES 5 with a textural class of clay, while only AES 3 has a clay loam textural class. The result was consistent with (Teferi et al., 2016) study on the Jedeb watershed that found altitude significantly affects the silt content, and the upland part (mean = 40.5%) was significantly higher than that of the midland part (mean = 26.9%).

Correspondingly, the soils at the upper landscape position of the watershed had a high mean value of silt (32.86%). In comparison, the lower landscape position had the lowest (21.73%), and the soils in the middle landscape had an intermediate (21.83%) silt content (Table 2). In contradiction to this result, Amuyou and Kotingo (2015) and Bufebo et al. (2021) found the highest and lowest mean value of silt content recorded in the lower and upper positions, respectively. Miheretu and Yimer (2018) found that variations in the silt content of the soil were not significant among landscape positions of the Gelana sub-watershed, Northern highlands of Ethiopia.

### 4.2 pH of the soil

The result shows that alkaline soil was found in the lower part of AES 1, according to Landon's (1991) rating. The high calcium carbonate content of the soil within the area of AES 1 of the Choke watershed affected the pH of the soil. In addition, soil erosion affects the concentration of pH in AES 1 of the Choke Mountain watershed. Since AES 1 is a lowland part of the watershed, soil was leaching from the upper into the lower part containing nutrients, making the soil alkaline. The lowest mean value (mean = 4.84) with the high acidity of soil pH was reported in the upper position of sloppy midland land (AES 4) (Table 7). It might be attributed to leaching off base ions (Ca, Mg, and K) because of high erosion in AES 4. There is a high mean annual rainfall (1200–1400 mm) in AES 4 that contributes to the leaching of bases.

According to Landon (1991), AES 4 and AES 3 ranged as strongly acidic, and AES 5 was moderately acidic soil attributed to variations in the intensity of tillage and climate. Similarly, Teferi et al. (2016), who conducted the study in the Jedeb watershed of the Blue Nile, also reported the mean pH value of the two agroecological zones (upper and midland) showed strongly acidic soil in the study area. Results from the study of Al Alwani and Al-Shaye (2019) also showed that the pH of the soil is determined by the soil's original material, the type of vegetation, and the climate (especially the amount of precipitation).

As far as the main effect of topo-sequence on soil acidity is concerned, a significant mean difference was found in the lower slope to the middle slope (mean difference = 0.698) and lower slope to the upper position of the watershed (mean difference = 0.755). The result suggests that pH increases as the slope of the landscape decreases. The lowest pH in the soils of the upper landscape position might be because of the loss of exchangeable bases caused by runoff and erosion and accumulated on the lower slope. These conditions increase the activity of hydrogen ions in the soil and reduce the soil pH. Studies by Bufebo et al. (2021), Dessalegn et al. (2014), and Miheretu and Yimer (2018) are like the current study, which reported that the mean value of soil pH is higher in the lower slope position than in the upper landscape. However, the study contradicts previous studies by Amuyou and Kotingo (2015), which showed a relatively high soil pH value in the upper slope segment compared to other parts of the catena. Mengistu et al. (2016) found a non-significant variation in soil pH among landscape positions. This contradiction could be attributed to variations in climatic conditions in the areas. Unless appropriate soil and water conservation measures are taken in this area, acidity might cause soil degradation, and sustainable crop productivity will be affected later.

### 4.3 Available phosphorus

The result of MANOVA showed available P varied substantially with AES and landscape position and the interaction effect of AES and landscape position (AES∗topo-sequence), with an effect size of 67%, 45%, and 56%, respectively (Table 6). The significant interaction effect of the two factors suggests that the effect of landscape position on available P depends on AES of Choke Mountain watershed. In each AES of the Choke, the farming system and input usage, including the application of DAP fertilizer, are different that determine the concentration of available P in the different topographic positions. In addition, the topographic positions of each AESs also have a substantial effect on available P that might contribute to the strong interaction effects. AES affected by the soil's acidity (AES 3 and AES 4) recorded the lowest mean value of available P (3.0 mg/kg and 3.18 mg/kg), respectively (Table 7). The result came from the positive correlation between soil pH and available P (*r* = 0.568) (Table 4). Al Alwani and Al-Shaye (2019) also reported that soil pH affected available nutrients, especially phosphorus.

A significant mean difference in available P was observed among the AES of the watershed. Soil with >25, 18–25, 10–17, 5–9, and 5 mg/kg, according to FAO (1980) available P ratings, is classified as very high, high, medium, low, and very low, respectively. Only AES 1 of the Choke has medium available P, whereas AES 4 and AES 5 have low available P, and AES 2 and AES 3 have deficient available P, according to these rates. The very low rate of available P was assigned to the middle landscape positions.

The lower and upper parts of topo-sequences have a low rate of available P (Table 2). Earlier researchers like Aytenew (2015) concluded that the variation of available P is highly associated with the variation of organic matter content in each slope gradient. However, the result showed that the available P content of soils did not decrease with a decrease in organic matter. In contradiction to these findings, available P was not statistically significant along with the various landscape positions (Amare et al., 2013; Anwanane et al., 2017; Mengistu et al., 2016).

### 4.4 Organic matter

The result shows that OM concentration increased from the lowland of the Abay gorge to the mountainous highlands of the Choke Mountain watershed. In agreement with this study (Abebe et al., 2020; Ebabu et al., 2020; Kidanemariam et al., 2012), reported higher OM content in higher altitudes than in lower elevations. Teferi et al. (2016) stated that the higher content of OM was in the middle part of the Jedeb watershed compared to the upper part of the area, which was attributed to different rates of SOM decomposition in the upland and midland of the watershed. According to the rating system established by Tadesse et al. (1991), the organic matter content of AES was categorized as low (AES 1 & 2), medium (AES 3 & 4), and high (AES 5).

Although the effect size was small based on Cohen's (1988) table, the topographic position also affects the distribution of SOM content in the Choke watershed. In line with this result, Amare et al. (2013), Bufebo et al. (2021), and Miheretu and Yimer (2018) reported the significant effect of landscape position on OM in their study area. The highest mean value of OM is concentrated in the upper position of the AES. The lowest mean value was on the middle slope, followed by the lower slope of the watershed (Table 2). Contrary to this study (Jendoubi et al., 2019), reported that higher OM was observed in the lower landscape because of erosion and deposition and lower SOM concentration in the upper position of the Mediterranean landscape. Besides, Aytenew (2015) found that the minimum OM was recorded in sloping and moderately steep soils. In contrast, the maximum OM was recorded in the soils of the gently sloping area. The effect of the slope gradient on the soil moisture storage capacity and biomass production, which is high in gently sloping areas, could contribute to the variation.

The difference in soil OM across the five AES and three landscape positions in the watershed could also be responsible for the local climate, land use, land management, and dominant soil type of the specific area. In line with this study, Abate et al. (2016) also show that the most probable sources of variation in SOM content among the land units might be variation in altitude, the intensity of cultivation, cropping system, and soil management practices. The low temperatures (7.5 °C–10 °C) of Choke AES 5 slow down the decomposition of SOM and create conditions of poor aeration (slow oxidation) (Bewket and Stroosnijder, 2003). The high temperature prevailing in AES 1 and AES 2 of Choke handles the rapid burning of OM, thus resulting in the low content of OM in those areas. In terms of land use and farming, intensive cultivation was practiced in AES 2 of Choke with high utilization of inorganic fertilizer but not in AES 5, which is inappropriate for high-intensity agriculture. In addition, in AES 5 of Choke, most farmers use organic fertilizer instead of chemical fertilizer as their local agricultural development agent. This shows that using organic materials helps enhance the soil's organic content, which will help to improve soil quality.

The dominant soil type existing in the AES of the Choke watershed plays a major role in the concentration of OM. The study on AES analysis of the Choke Mountain watershed by (Simane et al., 2013) showed that AES 2 is characterized by extensive areas of vertisol. Such soils are prone to severe waterlogging (soil compaction) during the rainy season. Although the BD of the soil was not significant in topo-sequence, agroecosystem, and their interaction effect, the highest (1.4 g/cm^3^) value of bulk density was found in AES 2. Whereas the lowest BD was recorded in AES 5 (0.7 g/cm^3^), showing the effect of soil compaction on OM in the study area. Pearson correlation coefficients also revealed a negative and significant correlation between OM and BD (*r* = -0.688) (Table 4).

Similarly, studies conducted in the southern Mediterranean highlands of Turkey reported that high OM in the soil makes it loose and porous, reducing its bulk density (Celik, 2005). Thus, the study suggests a proper technology that will prevent waterlogging and improve drainage in AES 2. Reducing tillage intensity and applying integrated, multifunctional cropping rotations that include forage legumes and small grains will improve SOM and soil quality.

### 4.5 Cation exchange capacity

The soil CEC was insignificant in the topo-sequence and interaction effects (topo-sequence∗ AES). However, the variation of CEC was statistically significant with a large effect size of (*η* ^*2*^ = 0.66) across the five AES of the Choke Mountain watersheds. The highest mean value of CEC was found in AES 2 (Table 2), where the dominant soil type was vertisol, which is valuable soil for cultivation. However, the major problems with this soil are low in OM concentration and soil compaction (waterlogging). In agreement with this study, Assefa (2015) also found vertisols of Gimbichu Woreda in the Oromia Region, which is level to gently sloping, has high CEC and nutrient availability but with the most severe problems of poor drainage, low OM, and total nitrogen. According to Landon (1991), topsoil with a CEC of >25, 15–25, 5–15, and <5 meq/100g is classified as high, medium, low, and very low. Based on these ratings, the mean value of CEC in all AES of the Choke Mountain watershed is categorized as high. Soil texture is one parameter that significantly influences CEC values (Tomašić et al., 2013). The textural classes of AES of the watershed soils vary from clay, clay loam, and loam to land use and management practice, topographic positions, parent materials, and clay minerals. Clay and OM are also the primary sources of soil CEC and are controlled by them (Aytenew, 2015; Rezaei et al., 2015).

Therefore, the distribution of CEC in different AES of Choke should be followed by clay and OM distribution patterns. The result, however, does not show a similar distribution pattern to clay. The Pearson correlation coefficient revealed that the CEC of the soil was negatively and significantly correlated with the clay content of the soil (*r* = -0.333) (Table 4). However, the results contradicted the results of Guteta and Abegaz (2017), Aytenew (2015), and Yitbarek et al. (2013), who reported a positive and significant correlation between CEC and the clay content of the soil.

## 5 Conclusion

The minimum datasets from fourteen soil quality indicators were selected using PCA. Silt content of the soil, soil pH, available P, OM, and CEC were selected as an MDS to evaluate the AES of Choke at various topo-units. The interaction of AES and topo-sequence (AES∗topo-sequence) affects soil silt content, pH, and available P, showing the impact of one independent variable depends on the other. The silt content of the soil was highest in the upper part of AES 5 and lowest in the lower position of AES 2. The upper position of AES 3 in the watershed was strongly acidic, attributed to leaching off base ions. AES 1 in the lower part recorded the highest pH mean value, which is slightly alkaline soil. A similar result was observed for available P, showing a positive correlation with soil pH.

Both AES and topo-unit uniquely affected the OM content of the soil, being higher in AES 5 and lower in AES 1. The variation might be attributed to climate, farming system, land use, land cover, and management practices, especially the tillage intensity across the five AES. The soil's CEC was relatively higher in AES 2. However, all AES were still under the higher range of CEC. Soil quality indicators varied along with topo-units and across the five AES of the Choke Mountain watershed. This finding suggests that farmers should be given suggestions for soil management and agronomic practices that are based on AES and consider specific topographic regions. As a result, the study recommends an immediate soil and water conservation plan for areas affected by soil acidity caused by leaching. For acid soils, calcium carbonate liming is recommended to boost agricultural production. Reduced tillage intensity and the use of integrated, multifunctional agricultural rotations that contain forage legumes will increase SOM and improve soil quality and the environment for soils afflicted by soil compaction and low OM content.

## Declarations

### Author contribution statement

Demeku Mesfin: Conceived and designed the experiments; Performed the experiments; Analyzed and interpreted the data; Contributed reagents, materials, analysis tools or data; Wrote the paper.

Engdawork Assefa & Belay Simane: Conceived and designed the experiments; Performed the experiments; Contributed reagents, materials, analysis tools or data.

### Funding statement

This work was supported by Addis Ababa University and Wolaita Sodo University.

### Data availability statement

Data will be made available on request.

### Declaration of interests statement

The authors declare no conflict of interest.

### Additional information

No additional information is available for this paper.

## Footnotes

1 The third-level of the administrative division of Ethiopia – after zones and the regional states.

2 The smallest administrative unit in Ethiopia.
